# *BRCA* testing, treatment patterns and survival in platinum-sensitive recurrent ovarian cancer – an observational cohort study

**DOI:** 10.1186/s13048-016-0227-x

**Published:** 2016-03-22

**Authors:** Sudhir K. Unni, Marisa B. Schauerhamer, Rishi Deka, Jerzy E. Tyczynski, Ancilla W. Fernandes, Vanessa Stevens, Diana I. Brixner, David D. Stenehjem

**Affiliations:** Department of Pharmacotherapy, Pharmacotherapy Outcomes Research Center (PORC), College of Pharmacy, University of Utah, 30 South 2000 East, Rm 4834 (4th Floor), Salt Lake City, UT 84112 USA; AstraZeneca Pharmaceuticals, Gaithersburg, MD USA; Program in Personalized Health Care, University of Utah, Salt Lake City, UT USA; Huntsman Cancer Institute, Salt Lake City, UT USA

**Keywords:** Platinum-sensitive ovarian cancer, *BRCA* testing, Survival, Systemic treatment

## Abstract

**Background:**

Breast cancer associated (*BRCA*) genes are critical for DNA repair. Mutations in *BRCA1* and *BRCA2* (*BRCA*m) result in loss of these repair mechanisms and potential carcinogenesis. Germline *BRCA*m are common in ovarian carcinomas, particularly in platinum-sensitive disease. The increased prevalence of *BRCA*m in platinum-sensitive disease is likely due to enhanced responsiveness to platinum chemotherapy from homologous recombination repair deficiency. The purpose of this study was to explore *BRCA* testing, treatment patterns and survival in platinum-sensitive recurrent (PSR) ovarian cancer.

**Methods:**

This was an observational cohort analysis of PSR ovarian cancer treated at the Huntsman Cancer Institute from 1995 to 2012. Germline *BRCA* status was ascertained through chart review and categorized as *BRCA*m (*BRCA1/2* positive), *BRCA*wt (*BRCA* wild type or variant of uncertain significance), and untested. Treatment patterns and survival were assessed from recurrence until death or last follow-up. The Kaplan-Meier method was used to evaluate survival from recurrence by *BRCA* status. Logistic regression and COX proportional hazard model was used to estimate predictors of *BRCA* testing and survival, respectively.

**Results:**

Of the 168 PSR patients, 15 (9 %) were *BRCA*m, 25 (15 %) were *BRCA*wt, and 128 (76 %) were untested. Median age at PSR was 56 years for *BRCA*m and *BRCA*wt (*p =* 0.90) and 63 years for those untested (*p =* 0.033 vs *BRCA*m). Overall survival was similar between *BRCA*m and *BRCA*wt (median 50.4 vs 67.5 months, *p =* 0.86) and was 24.9 months in untested patients. Significant predictors for the likelihood of *BRCA* testing were age (OR = 0.93, 95 % CI 0.89, 0.97, *p* = 0.002), family history of breast or ovarian cancer (OR = 8.33, 95 % CI: 3.08, 22.59, *p* < 0.001), and cancer diagnosis year (OR = 10.02, 95 % CI: 3.22, 31.21, *p* < 0.001). *BRCA*-tested patients had a lower risk of death versus untested (HR 0.35, 95 % CI 0.17, 0.68, *p* = 0.001).

**Conclusions:**

*BRCA*wt patients had similar outcomes to *BRCA*m patients, potentially owing to similar age at diagnosis, representing a *BRCA* testing channeling bias. Younger patients, those with a family history of breast or ovarian cancer, and those diagnosed more recently were more likely to be *BRCA* tested. BRCA tested patients had a lower risk of death.

**Electronic supplementary material:**

The online version of this article (doi:10.1186/s13048-016-0227-x) contains supplementary material, which is available to authorized users.

## Background

It is estimated that in 2015, there will be 21,290 new cases of ovarian cancer diagnosed in the United States [1]. While this type of cancer is rare compared with other types, the percentage of patients surviving 5 years after diagnosis is only 45.6 % [1].

The current standard of care for late-stage ovarian cancer is cytoreductive surgery followed by 6–8 cycles of combination chemotherapy with a platinum-containing agent such as carboplatin [2]. Patients who respond to platinum-based therapy and experience a relapse of ovarian cancer greater than 6 months after treatment completion are considered to have platinum-sensitive ovarian cancer [3]. Those who experience a relapse during treatment or within 6 months after treatment are considered to have platinum-resistant ovarian cancer [4] and are unlikely to respond to additional platinum-based treatments. However, the majority of patients at first recurrence of ovarian cancer have platinum-sensitive disease [3] and standard therapy in these patients consists of retreatment with a platinum-containing regimen [2]. It has also been found that deleterious mutations in *BRCA* (breast cancer associated) genes are more prevalent in patients with platinum-sensitive ovarian cancer compared with platinum-resistant disease [5]; this finding has implications for improving the treatment of recurrent ovarian cancer.

*BRCA1* and *BRCA2* are genes that are critical for DNA repair through homologous recombination [6]. Germline and somatic mutations in either *BRCA1* or *BRCA2* result in a significant increase in genomic instability and errors leading to carcinogenesis due to homologous recombination repair deficiency [7]. Loss of function in both genes results in cell death, whereas loss of function in one gene allows for survival of cells with faulty DNA repair mechanisms [8]. Therefore, patients with germline *BRCA1/2* mutations (*BRCA*m) have enhanced susceptibility to agents that target DNA, such as platinum-containing agents, because of induction of synthetic lethality [5, 9–11].

Owing to the enhanced susceptibility of platinum-containing agents in *BRCA*m platinum-sensitive recurrent ovarian cancer and the prevalence of *BRCA*m in recurrent disease, it is important to evaluate *BRCA* testing, treatment patterns and survival in patients who may benefit most from platinum therapy. The purpose of this study was to assess *BRCA* testing patterns, treatment patterns, and survival in patients with platinum-sensitive recurrent ovarian cancer.

## Methods

### Study design and data source

This was an observational cohort study of women with platinum-sensitive recurrent (PSR) ovarian cancer treated at the Huntsman Cancer Institute (HCI) in Salt Lake City, Utah. The Huntsman Cancer Institute tumor registry (HCI-TR) was used to identify patients with site and histology codes for epithelial ovarian cancer between January 1, 1995 and December 31, 2012. Identified patients were linked to electronic health record data from the University of Utah Enterprise Data Warehouse (EDW), which provided patient demographic and clinical information, including laboratory test results, medications, procedures, health status and physician notes.

### Study population

Included patients were 18 years and older, with a diagnosis of epithelial ovarian cancer (ICD-10/ICD-O C56.9), fallopian tube cancer (C57.0), or primary peritoneal cancer (C48.1–48.3) in the HCI-TR, and had at least two health care visits separated by ≥30 days with ICD-9 codes for ovarian (183.X), fallopian tube (183.2) or primary peritoneal cancer (158.x) at HCI. Patients who received a platinum-based regimen (carboplatin or cisplatin) for initial systemic treatment of ovarian, fallopian tube, or primary peritoneal cancer and had a platinum-free interval (PFI) of at least 6 months before detection of recurrence were identified in the EDW or via chart review, classified as PSR ovarian cancer patients and included in the final analysis. Patients were excluded if they had a diagnosis of *in situ* ovarian cancer, had no documented use of platinum-containing agents, were platinum refractory, had a PFI of <6 months, or had no evidence of recurrence. The date of the first recurrence in the EDW was considered the index date for time to event data. The HCI-TR provided the date, stage, pathology, histological grade, and primary tumor site at ovarian cancer diagnosis. Clinical characteristics such as family history of hereditary breast or ovarian cancer and personal history of breast cancer were assessed at recurrence using chart review in the EDW. The University of Utah’s Institutional Review Board and the HCI Clinical Cancer Investigations Committee approved this study.

### Classification of platinum-sensitive recurrent ovarian cancer

All patients who received platinum-based first-line treatment were identified in the EDW or via chart review and assessed for response and the PFI. PFI was defined as the number of months from the last platinum dose to recurrence. Those without evidence of recurrence after first-line treatment were excluded. Patients relapsing during first-line treatment or within 6 months of the last platinum treatment were categorized as platinum refractory and also excluded. Platinum sensitivity was defined as a response to first-line platinum treatment and a PFI of ≥6 months or physician-documented platinum sensitivity.

### *BRCA* testing and status

*BRCA* status was ascertained through chart review. All medical records with mention of BRCA were reviewed by a semi-automated keyword search of the electronic notes with a custom text search tool that searched keywords or patterns of words using Boolean constructs. Patients were classified as *BRCA*m (*BRCA1*/*2* positive) if a deleterious *BRCA1* or *BRCA2* germline mutation was documented in the medical record. Patients were classified as *BRCA*wt (*BRCA* wild type or variant of uncertain significance) if *BRCA* testing was conducted and documentation of the wild-type *BRCA* gene (no deleterious mutation detected) or a variant of uncertain significance was recorded. Lastly, patients were classified as untested if *BRCA* testing was not performed or if *BRCA* status was not recorded in the medical record.

### Treatment patterns

Primary treatment modalities at the time of ovarian cancer diagnosis were categorized as systemic chemotherapy, radiation, or cytoreductive surgery; these treatments were also evaluated from recurrence until death or last follow-up. Systemic chemotherapy was further categorized as first-line, second-line, and third-line treatment based on the treatments received from recurrence as assessed by chart review. Systemic treatment lines after the initial treatment at recurrence were defined as a change in systemic treatment (addition and/or deletion of drug) due to disease progression, adverse events, or tolerability. Retreatment with the same systemic treatment after a delay in treatment as a result of an adverse event or the inability to tolerate the regimen was not considered a new treatment line. In patients who had a complete response to treatment and in whom systemic treatment was subsequently discontinued, an additional treatment line was considered if the same or alternative systemic treatment was restarted at subsequent disease recurrence/progression.

### Statistical analysis

Descriptive statistical analyses, including mean and standard deviation for continuous variables and count and percentage for categorical variables, were performed. Student *t*-test and Wilcoxon rank-sum test were used for continuous variables and Fisher’s exact test was used for categorical variables. A significance level of 5 % was utilized for this study.

Logistic regression was conducted to estimate the likelihood of patients receiving *BRCA* testing (*BRCA*m or *BRCA*wt) versus untested patients. The covariates in the model included age at ovarian cancer diagnosis, ethnicity, family history of breast or ovarian cancer, personal history of breast cancer, ovarian cancer diagnosis stage, pathology, primary tumor site, and year of ovarian cancer diagnosis.

Kaplan-Meier methodology was used to evaluate survival from index date (date of PSR) until death or last follow-up. Survival was stratified by *BRCA* status and compared by log-rank test. A Cox proportional hazard model was constructed from recurrence to death or time of last follow-up, whichever occurred earlier, by including patient demographics, clinical characteristics, family history or personal history of breast or ovarian cancer, year of ovarian cancer diagnosis, and *BRCA* testing status (tested vs. untested) as covariates in a single model run.

## Results

### Patient characteristics

**Fig. 1:**
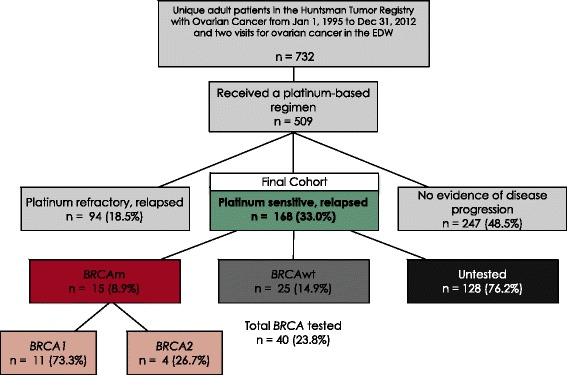
Patient Selection and *BRCA* Status

There were 732 unique adult patients identified in the HCI-TR with an ovarian cancer diagnosis between January 1, 1995 and December 31, 2012 with at least two visits for ovarian cancer in the EDW (Fig. 1). Of those diagnosed with ovarian cancer, 509 patients received a platinum agent, and of those, 168 (33 %) had documented PSR disease (final study cohort), 94 (18 %) had relapsed, platinum-refractory disease, and 247 (49 %) had no evidence of disease progression.

### *BRCA* testing and mutational status

**Fig. 2:**
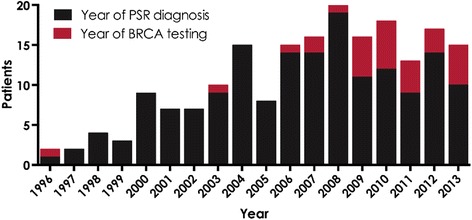
Number of Patients by Year of PSR Diagnosis and Year of *BRCA* Testing

Of the 168 PSR patients, 15 (9 %) had *BRCA*m, 25 (15 %) had *BRCA*wt, and 128 (76 %) were classified as untested. Of the 15 *BRCA*m patients, 11 (73 %) had mutations in *BRCA1* and four had mutations in *BRCA2. BRCA* testing was predominantly performed from 2006 to 2013, and only two patients received testing prior to 2006 (Fig. 2).

### Demographic and clinical characteristics

Median age at recurrence was 58 years (interquartile range [IQR]: 48–64) for *BRCA*m, 57 years (IQR: 50–63) for *BRCA*wt and 63 years (IQR: 54–71) for untested patients, Table 1. More than 50 % of the study patients were white (Table 1). *BRCA* tested patients had a higher percentage of those with a family history of breast or ovarian cancer compared to untested patients (65 % vs 20 %, *p* < 0.001). *BRCA* tested patients also had significantly higher rate of a personal history of breast cancer than untested patients (20 % vs. 5 %, *p* = 0.009) (Table 1), however a statistical difference was not observed between *BRCA*m and *BRCA*wt (33 % vs. 12 %, *p =* 0.13). There were no significant differences (*p* < 0.05) between the comparison groups for other clinical variables.

**Table 1:** Demographics and Clinical Characteristics of Study Patients (*N* = 168)

	*BRCA*m *n* = 15	*BRCA*wt *n* = 25	*p*-Value*	Untested *n* = 128	*p*-Value**
Year of Ovarian Cancer Diagnosis					
Median (IQR)	2007 (2004–2009)	2008 (2005–2010)	0.305	2004 (2000–2007)	**0.027**
Year of PSR Diagnosis					
Median (IQR)	2008 (2006–2011)	2010 (2008–2012)	0.201	2006 (2003–2009)	**0.043**
Age at Recurrence, Years					
Median age (IQR)	58 (48–64)	57 (50–63)	0.900	63 (54–71)	0.052
Mean age ± SD	56 ± 11.0	56 ± 9.7	0.903	63 ± 12.7	**0.033**
Age group					
<60 years	8 (53.3 %)	15 (60.0 %)	0.231	50 (39.1 %)	**0.039**
60–74 years	5 (33.3 %)	10 (40.0 %)		55 (43.0 %)	
≥75 years	2 (13.3 %)	0		23 (18.0 %)	
Ethnicity					
White	8 (53 %)	18 (72 %)	0.138	84 (66 %)	0.372
Hispanic	2 (13 %)	0 (0 %)		7 (5 %)	
Other	0 (0 %)	2 (8 %)		6 (5 %)	
Unknown	5 (33 %)	5 (20 %)		31 (24 %)	
Family History of Hereditary Breast or Ovarian Cancer					
No family history	0 (0 %)	14 (56 %)	**<0.001**	103 (80 %)	**<0.001**
Familial risk	15 (100 %)	11 (44 %)		25 (20 %)	
Personal History of Breast Cancer					
No history	10 (67 %)	22 (88 %)	0.126	121 (95 %)	**0.003**
History of breast cancer	5 (33 %)	3 (12 %)		7 (5 %)	
Stage at Ovarian Cancer Diagnosis					
≤2	2 (13.3 %)	5 (20 %)	0.900	21 (16.4 %)	0.917
≥3	10 (66.7 %)	15 (60 %)		87 (68.0 %)	
Unknown	3 (20 %)	5 (20 %)		20 (15.6 %)	
Pathology					
Serous	10 (66.7 %)	18 (72 %)	0.910	77 (60.2 %)	0.759
Adenocarcinoma	1 (6.7 %)	1 (4 %)		14 (10.9 %)	
Endometrioid	0	1 (4 %)		11 (8.6 %)	
Other	4 (26.7 %)	5 (20 %)		26 (20.3 %)	
Tumor Histologic Grade at Ovarian Cancer Diagnosis					
1 and 2	2 (13.3 %)	3 (12 %)	0.847	15 (11.7 %)	0.855
3	7 (46.7 %)	8 (32 %)		68 (53.1 %)	
4	2 (13.3 %)	5 (20 %)		12 (9.4 %)	
Unknown	4 (26.7 %)	9 (36 %)		33 (25.8 %)	
Primary Tumor Site					
Ovary	12 (80 %)	20 (80 %)	0.574	113 (88.3 %)	0.363
Peritoneum	3 (20 %)	3 (12 %)		11 (8.6 %)	
Fallopian tube	0 (0 %)	2 (8 %)		4 (3.1 %)	
Ca-125 at Recurrence (Highest Value ±30 days), U/mL					
Median Ca-125 (IQR)	195 (82–274)	49.5 (36.5–178)	0.218	90 (34–343)	0.549
ECOG score					
Median ECOG (IQR)	1 (0–1)	0 (0–1)	0.457	1 (0–1)	0.634
ECOG group					
≤2	6 (40 %)	14 (56 %)	0.514	34 (26.6 %)	0.490
≥3	0	0		2 (1.6 %)	
Unknown	9 (60 %)	11 (44 %)		92 (71.9 %)	
Initial Treatment at Ovarian Cancer Diagnosis					
Chemotherapy	7 (46.7 %)	12 (48 %)	0.547	50 (39.1 %)	0.504
Neoadjuvant chemotherapy	4 (26.7 %)	3 (12 %)		11 (8.6 %)	
Radiation therapy	0	0		2 (1.6 %)	
Primary debulking or cytoreductive surgery	9 (60 %)	23 (92 %)		110 (86 %)	
Missing	0	0		1 (0.8 %)	
Duration of Follow-up, Days					
Median (IQR)	1064 (656–2231)	981.5 (434–1546)	0.419	490 (219–1015)	0.005

*BRCAm vs. *BRCA*wt Fisher’s exact test for categorical variables, Wilcoxon rank-sum or *t*-test for continuous variables; **BRCAm vs. Untested Fisher’s exact test for categorical variables, Wilcoxon rank-sum or *t*-test for continuous variables

The median time from ovarian cancer diagnosis to PSR disease was 20.1 months (*BRCA*m), 22.4 months (*BRCA*wt), and 19.0 months (untested) and was not significantly different between the comparison groups (*BRCA*m vs. *BRCA*wt, *p* = 0.850) (Additional file 1: Table S1). Similarly, the median PFI was 12.1 months (*BRCA*m), 14.5 months (*BRCA*wt), and 13.1 months (untested) and was not significantly different between the comparison groups (*BRCA*m vs. *BRCA*wt, *p* = 0.960) (Additional file 1: Table S1).

### Treatment patterns

**Fig. 3:**
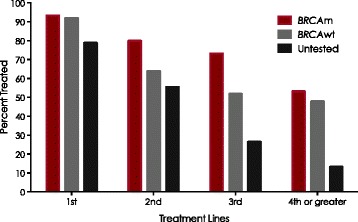
Systemic Treatment Lines Received for Platinum-Sensitive Recurrence Date

Systemic treatment, including chemotherapy and hormonal agents, was administered in the majority of patients at recurrence (93.3 % *BRCA*m, 92.0 % *BRCA*wt, and 78.9 % untested, Table 2). Secondary cytoreductive surgery was performed in 46.7 % (*n* = 7) of *BRCA*m, 52 % (*n* = 13) of *BRCA*wt, and 40.6 % (*n* = 52) of untested patients. Radiation therapy was used more frequently in the *BRCA*m (*n* = 7, 46.7 %) and *BRCA*wt (*n* = 9, 36 %) patients compared with untested patients (*n* = 25, 19.5 %). All patients in the *BRCA*m and *BRCA*wt groups received at least one treatment (systemic, cytoreductive surgery or radiation therapy). However, in the untested group, 2.3 % of patients (*n* = 3) received no treatment and 3.9 % of patients (*n* = 5) had unknown/missing treatments (Table 2). The proportion of patients who received a platinum-containing regimen any time after recurrence was 87 % (*n* = 13) in the *BRCA*m, 76 % (*n* = 19) in the *BRCA*wt, and 67 % (*n* = 118) in untested group. No statistical difference in the number of treatment lines were observed between *BRCA*m and *BRCA*wt groups (median 4 BRCAm vs. 3 BRCAwt, *p =* 0.83). The median number of systemic treatment lines was two in the untested group (Fig. 3).

**Table 2:** Systemic Treatment, Surgery, Radiation and Utilization Post-Recurrence

Treatment Type	*BRCA*m			*BRCA*wt			Untested		
	*n* = 15	% or Range	*p*-Value	*n* = 25	% or Range	*p*-Value*	*n* = 128	% or Range	*p*-Value**
Systemic Treatment									
Systemic treatment received, n	14	93.3 %	ref	23	92.0 %	0.876	101	78.9 %	0.138
Missing/unknown, n	1	6.7 %	-	1	4.0 %	-	16	12.5 %	-
Median number of treatment lines (IQR)	4 (2–4)	0–6	ref	3 (1–5)	0–11	0.832	2 (1–3)	0–6	**0.001**
Platinum-containing regimen post-recurrence, n	13	86.7 %	ref	19	76.0 %	0.404	86	67.2 %	0.098
Cytoreductive Surgery									
Secondary cytoreductive surgery, n	7	46.7 %	ref	13	52.0 %	0.744	52	40.6 %	0.655
Missing/unknown, n	0	0.0 %	-	0	0.0 %	-	5	3.9 %	-
Median months to cytoreductive surgery from recurrence (IQR)	8.8 (1.5–38.8)	0–93.5	ref	0.8 (−0.4–3.4)	−0.5–11.7	0.067	0.4 (−0.1–3.4)	−0.8–71.9	0.064
Radiation Therapy									
Radiation therapy, n	7	46.7 %	ref	9	36.0 %	0.506	25	19.5 %	**0.027**
Missing/unknown, n	0	0.0 %	-	0	0.0 %	-	5	3.9 %	-
Median months to radiation therapy from recurrence (IQR)	15.8 (7.2–21.9)	1.7–29.4	ref	19.7 (17.0–26.5)	11.7–34.5	0.333	3.5 (1.3–11.7)	0.1–81.5	0.109
No Treatment (Surgery, Radiation, Systemic), n	0	0.0 %	-	0	0.0 %	-	3	2.3 %	-
Total Unknown/Missing (Surgery, Radiation, Systemic), n	0	0.0 %	-	0	0.0 %	-	5	3.9 %	-

*BRCAm vs. *BRCA*wt;**BRCAm vs. Untested

### Survival analysis

**Fig. 4:**
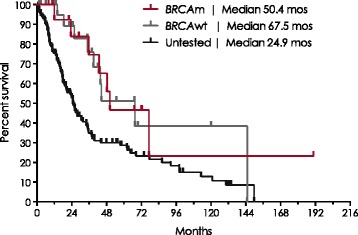
Kaplan-Meier Survival Estimates from Platinum-Sensitive Recurrence Date

The overall survival for patients with a BRCAm was similar to the BRCAwt group (log rank, *p* = 0.855), Fig. 4 and Additional file 1: Table S2. Median overall survival from recurrence was 50.4 months in BRCAm, 67.5 months in BRCAwt and 24.9 months in the untested group.

### Predictors of *BRCA* testing

The significant predictors for the likelihood of *BRCA* testing were age (decreased likelihood with a 1-year increase in age, odds ratio [OR] = 0.93, 95 % CI: 0.89, 0.97, *p* = 0.002), family history of breast or ovarian cancer (increased likelihood with positive history, OR = 8.33, 95 % CI: 3.08, 22.59, *p* < 0.001), and year of ovarian cancer diagnosis (increased likelihood after 2006, OR = 10.02, 95 % CI: 3.22, 31.21, *p* < 0.001) (Additional file 1: Table S3).

### Predictors of survival from platinum-sensitive recurrence

Patients who received a *BRCA* test had a lower risk of death than untested patients (hazard ratio [HR] = 0.35, 95 % CI 0.17, 0.68, *p* = 0.001). Also, the risk of death increased by 2 % for each increasing year of age (HR = 1.020, 95 % CI 1.001, 1.040, *p* = 0.039) (Additional file 1: Table S4).

## Discussion

This study provides a unique perspective into *BRCA* testing, clinical characteristics, treatment patterns, and survival outcomes in unselected, consecutive patients with PSR ovarian cancer. These data suggest that tested patients are younger at diagnosis, receive more treatment lines and have improved survival compared with untested patients.

This study demonstrates that in an academic oncology center with extensive genetic services support, approximately 24 % (*n* = 40) of patients with PSR ovarian cancer (*n* = 168) were tested for *BRCA*m. Of those who were tested, 37.5 % (*n* = 15) tested positive for a deleterious mutation in *BRCA1* or *BRCA2*, indicating a high pre-test probability for *BRCA*m, since it is thought that only 12–15 % of invasive ovarian cancers are associated with *BRCA*m [12, 13]. The high probability for *BRCA*m was also supported by other factors such as family history of breast or ovarian cancer, which was observed in all *BRCA*m patients and is known to be positively associated with *BRCA* testing.

*BRCA1/2* testing was made commercially available in 1996 by Myriad Laboratories, Inc. Utilization of *BRCA* testing increased during the study period, with more patients with PSR ovarian cancer being *BRCA* tested after 2006, which is substantiated by outside reports of increased utilization of *BRCA* testing [14]. Also, it was in 2006 that HCI instituted a Hereditary Risk Evaluation Program, which may have contributed to increased *BRCA* testing in subsequent years and may account for the variation in the number of tested patients before and after 2006.

These data affirm that patients with a family history of hereditary breast or ovarian cancer are more likely to have been *BRCA* tested, with all *BRCA*m patients having a history compared with 44 % of *BRCA*wt and 20 % of untested patients. Additionally, *BRCA*m patients (33 %, *n* = 5) were more likely to have a personal history of breast cancer, either prior to ovarian cancer diagnosis or concurrently, compared to untested (5 %, *n* = 7) and *BRCA*wt patients (20 %, *n* = 3).

*BRCA*-tested patients were also significantly younger than untested patients. The median age for *BRCA*m patients was similar to those in other reports [15]. The similar age of *BRCA*m and *BRCA*wt patients potentially represents a channeling bias as patients diagnosed at a younger age are more likely have a *BRCA*m and clinicians would be more likely to offer genetic testing to younger patients based on guideline recommendations [2].

Overall, younger age at diagnosis of ovarian cancer, family history of hereditary breast or ovarian cancer, and personal history of breast cancer were all significant predictors of *BRCA* testing in this population and suggests that testing was motivated by genetic risk assessment. Additionally, based on other population-based studies indicating a *BRCA*m rate of 12–15 % [12, 13] in invasive ovarian cancer, our cohort would be expected to contain 20–25 *BRCA*m carriers. Therefore, over the study period, genetic risk assessment and *BRCA* testing potentially discovered 60–75 % of the expected *BRCA*m carriers. However, the expected *BRCA*m rate may be higher in patients with PSR ovarian cancer. Universal *BRCA* testing is now recommended for all patients with high-grade serous ovarian cancer and would have likely identified additional *BRCA* carriers [2].

Our study identified 11 *BRCA1* and 4 *BRCA2* mutation carriers thus a *BRCA1*-to-*BRCA2* ratio of ~3:1. There are geographic and ethnic differences in *BRCA1* and *BRCA2* mutations [16]. Common founder *BRCA1/2* mutations with a significant role in the population occur in Ashkenazi Jews, as well as in Iceland, Russia, Germany, Hungary, Norway, Finland, Sweden, Denmark, France, the Netherlands, and the UK [16]. Overall, 8–40 % of all *BRCA1* mutations have been identified in families from the UK, USA, France, Germany, Italy, and the Netherlands [16]. In 13 studies containing at least 60 families in which one or more cases of ovarian cancer were ascertained, the frequency of *BRCA1* ranged from 24.2 to 76.2 % and the frequency of *BRCA2* ranged from 1.0 to 16.7 % [16]. The overall ratio of *BRCA1* to *BRCA2* mutations ranged from 2:1 to 62:1 [16]. These 13 studies mostly took place in European countries (aside from Australia and the USA). Thus, the *BRCA1*: *BRCA2* ratio of 3:1 in our study is consistent given that the majority of Utah inhabitants are of Northern European descent.

Improved ovarian cancer survival outcomes in *BRCA*m carriers have been reported [15, 17]. The largest and most recent meta-analysis demonstrated that *BRCA1* and *BRCA2* mutations have positive prognostic effects on ovarian cancer overall and progression-free survival [18]. The results of this meta-analysis revealed that *BRCA1* mutation carriers were associated with better overall survival than non-carriers, with a pooled HR of 0.76 (95 % CI: 0.70, 0.83) [18]. However, *BRCA2* mutation carriers were associated with even better survival outcomes compared with non-carriers, with a pooled HR of 0.58 (95 % CI: 0.50, 0.66) [18]. Platinum-free survival was also improved in *BRCA1* mutation carriers (HR = 0.65; 95 % CI: 0.52, 0.81) and *BRCA2* mutation carriers (HR = 0.61; 95 % CI: 0.47, 0.80) [18]. Though we did not evaluate survival in *BRCA1* and *BRCA2* patients because of sample size limitations, median survival from recurrence was not significantly different between *BRCA*m and *BRCA*wt patients in our study, whereas it was lower in untested patients.

These data indicated that tested patients had better OS than untested patients. However, this result should be interpreted with caution as the tested patients tended to be younger and their diagnoses were more recent and thus changes in treatment practice may be contributing. Furthermore, these data support the necessity for testing patients to ensure early diagnosis and optimization of the treatment options.

Potentially contributing to the improved survival, tested patients also received more systemic treatment lines after diagnosis of PSR ovarian cancer than untested patients. However there were no significant differences between *BRCAm* and *BRCAwt* patients, potentially reflecting the lack of available targeted therapies. The proportion of patients who received a platinum-containing regimen at any time after PSR diagnosis was 87 % (*n* = 13) in *BRCAm*, 76 % (*n* = 19) *BRCAwt*, and 67 % (*n* = 118) in untested patients. Utilization of secondary cytoreductive surgery and palliative radiation therapy was similar between *BRCAm* (46.7 %) and *BRCAwt* (36 %) patients; however, fewer untested patients received palliative radiation therapy (19.5 %). The increased use of palliative radiation therapy in *BRCA* tested patients may be partially explained by their increased duration of survival and therefore increased opportunity to receive radiation therapy.

There are several limitations of this research that should be considered when interpreting the results of this study. These results were from a single institution and may not be generalizable to a larger population or geographic area where clinical practices differ. Furthermore, the demographic composition of the study resulted in under-representation of some demographic categories and small sample sizes for some groups, such as races other than Caucasian/white, precluding meaningful comparisons across some categories of interest. The practice patterns observed at the HCI, a National Cancer Institute Designated Center and member of the National Comprehensive Cancer Network, may not be typical of those in other types of treatment facilities and institutions.

Data from medical charts and tumor registries can be subject to missing data and coding errors. Although most of the demographic and clinical characteristics and treatment patterns of interest for this study were reported as known, there were several variables in the overall population with non-negligible missing or unknown values such as race/ethnicity, stage at ovarian cancer diagnosis, and ECOG performance status, which may have an impact on survival.

Lastly, this study was limited by the small number of *BRCA* tested patients (*n* = 40) versus the larger cohort of untested patients (*n* = 128) and in particular, the resulting small sample size of *BRCA*m carriers (*n* = 15) which did not allow for meaningful characterization of survival and treatment patterns between *BRCA1* and *BRCA2* mutation carriers. An inherent problem of observational studies is the possibility of selection bias; as this was an observational study of real-world *BRCA* testing practices, the *BRCA* test selection bias (younger age and more recent year of diagnosis) made it difficult to compare differences between *BRCA*m and *BRCA*wt patients. Also, owing to the limitations of available observational data, the untested group was used as a comparison group; this group was assumed to contain predominantly non-*BRCA*m carriers.

## Conclusion

This study demonstrates in an academic oncology center with extensive genetic services support that *BRCA* testing rates increased during the study period and that the likelihood of *BRCA* testing increased with lower age, positive family history, and presenting time. However, wider *BRCA* testing may have identified additional *BRCA* carriers. Patients tested for *BRCAm* had greater median overall survival rates versus untested patients and a corresponding greater number of systemic treatment lines. *BRCA*wt patients had similar outcomes to *BRCA*m patients. Overall, this study continues to demonstrate the distinct clinical behavior of *BRCA*-mutated ovarian cancer and underscores the importance of appropriate genetic risk assessment and *BRCA* testing.

## Statement of ethics approval

The study was approved by the Institutional Review Board of the University of Utah via IRB Number IRB_00068779 and the Huntsman Cancer Institute’s Clinical Cancer Investigations Committee.

## Consent to publish

This is not applicable here as all data included in this study that was extracted from the University of Utah Enterprise Data Warehouse (EDW) was de-identified without any personal health information. The data that was collected through patient chart reviews was also collected without any personal health information

## Availability of data and materials

The data for this study is housed in a secure dedicated database server in the Pharmacotherapy Outcomes Research Center at the University of Utah in Salt Lake City, Utah.

## Additional file

Additional file 1: Supplementary Tables and Figures. (DOCX 80 kb)

## Abbreviations

BRCA: breast cancer associated
BRCAm: breast cancer associated mutation
BRCAwt: breast cancer associated wild type or variant of uncertain significance
PSR: platinum-sensitive recurrent
