# Supplementary material for: BRCA testing, treatment patterns and survival in platinum-sensitive recurrent ovarian cancer – an observational cohort study
Source: J Ovarian Res. 2016 Mar 22;9:18. doi: 10.1186/s13048-016-0227-x (PMC4802840; doi:10.1186/s13048-016-0227-x)
Supplement: Additional file 1: — Supplementary Tables and Figures. (DOCX 80 kb) [file 13048_2016_227_MOESM1_ESM.docx]

**Supplementary materials**

**Table 1. Median Time from Diagnosis of Ovarian Cancer and from Last Dose of a Platinum-Containing Agent to PSR Ovarian Cancer**

|  | **n** | **Months from Diagnosis to Recurrence (n = 168)** | | **Platinum-free Interval (n = 152)** | |
| --- | --- | --- | --- | --- | --- |
|  |  | **Median (IQR)** | ***p*-Value*** | **Median (IQR)** | ***p*-Value*** |
| *BRCA*m | 15 | 20.1 (15.7–27.3) | ref | 12.1 (10.5–25.4) | ref |
| *BRCA*wt | 25 | 22.4 (14.6–29.3) | 0.850 | 14.5 (8.6–21.5) | 0.960 |
| Untested | 128 | 19.0 (14.4–28.5) | 0.780 | 13.1 (8.7–23.9) | 0.923 |

**BRCA*m vs. *BRCA*wt and *BRCA*m vs. untested. IQR = interquartile range

**Figure 1.** **Months from Ovarian Cancer Diagnosis to Platinum-Sensitive Recurrence Date**

**Figure 2. Platinum-free Interval**

**Table 2. Median Survival from Diagnosis of Ovarian Cancer and from Platinum-Sensitive Recurrence Date**

|  | **n** | **Number of Deaths** | **Median Survival from Recurrence (Months)** | ***p*-Value,**  **Log Rank** |
| --- | --- | --- | --- | --- |
| **Survival from Ovarian Cancer Diagnosis** | | | | |
| *BRCA*m | 15 | 7 | 80.9 | ref |
| *BRCA*wt | 25 | 9 | 118.6 | 0.996 |
| Untested | 128 | 94 | 49.1 | **0.049** |
| **Survival from Recurrence Date** | | | | |
| *BRCA*m | 15 | 7 | 50.4 | ref |
| *BRCA*wt | 25 | 9 | 67.5 | 0.855 |
| Untested | 128 | 94 | 24.9 | **0.027** |

**Table 3. Logistic Regression for the Likelihood of Ovarian Cancer Patients Receiving *BRCA* Testing (n = 168)**

| **Variable** | **Odds Ratio** | **95% CI**  **Lower** | **95% CI Upper** | ***p*-Value** |
| --- | --- | --- | --- | --- |
| **Ovarian Cancer Diagnosis Age** | | | | |
| Age increase of 1.0 year | 0.930 | 0.889 | 0.973 | **0.002** |
| **Ethnicity** | | | | |
| Caucasian | ref |  |  |  |
| Hispanic | 0.169 | 0.014 | 2.081 | 0.165 |
| Other | 1.493 | 0.194 | 11.457 | 0.700 |
| Unknown | 1.372 | 0.412 | 4.570 | 0.607 |
| **Family History of Breast or Ovarian Cancer** | | | | |
| Yes vs. no | 8.334 | 3.075 | 22.589 | **<0.001** |
| **Personal History of Breast Cancer** | | | | |
| Yes vs. no | 3.639 | 0.855 | 15.492 | 0.081 |
| **Ovarian Cancer Diagnosis Stage** | | | | |
| Stage 1 and 2 | ref |  |  |  |
| Stage 3 | 1.312 | 0.330 | 5.218 | 0.700 |
| Stage 4 | 0.883 | 0.157 | 4.971 | 0.888 |
| Stage unknown | 0.523 | 0.077 | 3.548 | 0.507 |
| **Pathology** | | | | |
| Adenocarcinoma | ref |  |  |  |
| Endometrioid | 0.220 | 0.007 | 7.407 | 0.399 |
| Other | 3.054 | 0.349 | 26.701 | 0.313 |
| Serous | 2.361 | 0.321 | 17.356 | 0.399 |
| **Primary Tumor Site** | | | | |
| Ovary | ref |  |  |  |
| Fallopian tube | 1.268 | 0.155 | 10.352 | 0.825 |
| Peritoneum | 3.935 | 0.562 | 27.564 | 0.168 |
| **Year of Ovarian Cancer Diagnosis** | | | | |
| After 2006 vs. before 2006 | 10.018 | 3.216 | 31.211 | **<0.001** |

**Table 4. Cox Proportional Hazard Model for Survival from Platinum-Sensitive Recurrence Date (n = 168)**

| **Variable** | **Hazard Ratio** | **95% CI**  **Lower** | **95% CI Upper** | ***p*-Value** |
| --- | --- | --- | --- | --- |
| ***BRCA* Testing** | | | | |
| Tested vs. untested | 0.353 | 0.174 | 0.677 | **0.001** |
| **Ovarian Cancer Diagnosis Age** | | | | |
| Age increase of 1.0 year | 1.020 | 1.001 | 1.040 | **0.039** |
| **Ethnicity** | | | | |
| Caucasian | ref |  |  |  |
| Hispanic | 1.746 | 0.671 | 6.017 | 0.275 |
| Other | 0.717 | 0.260 | 2.564 | 0.574 |
| Unknown | 0.939 | 0.597 | 1.511 | 0.791 |
| **Family History of Breast or Ovarian Cancer** | | | | |
| Yes vs. no | 1.439 | 0.858 | 2.367 | 0.165 |
| **Personal History of Breast Cancer** | | | | |
| Yes vs. no | 1.552 | 0.705 | 3.095 | 0.260 |
| **Ovarian Cancer Diagnosis Stage** | | | | |
| Stage 1 and 2 | ref |  |  |  |
| Stage 3 | 1.141 | 0.633 | 1.979 | 0.650 |
| Stage 4 | 0.662 | 0.345 | 1.241 | 0.199 |
| Stage unknown | 1.077 | 0.486 | 2.516 | 0.859 |
| **Pathology** | | | | |
| Adenocarcinoma | ref |  |  |  |
| Endometrioid | 1.164 | 0.455 | 3.147 | 0.755 |
| Other | 1.138 | 0.542 | 2.296 | 0.725 |
| Serous | 1.342 | 0.687 | 2.438 | 0.372 |
| **Primary Tumor Site** | | | | |
| Ovary | ref |  |  |  |
| Fallopian tube | 1.531 | 0.531 | 6.502 | 0.467 |
| Peritoneum | 1.322 | 0.511 | 3.499 | 0.564 |
| **Year of Ovarian Cancer Diagnosis** | | | | |
| After 2006 vs. before 2006 | 1.412 | 0.870 | 2.268 | 0.161 |
